# Psychometric Validation of the Connor–Davidson Resilience Scale 10 in Peruvian Nurses and Its Association with Stress and Empathy

**DOI:** 10.3390/healthcare14081097

**Published:** 2026-04-20

**Authors:** Roberto Zegarra-Chapoñan, Jhon Alex Zeladita-Huaman, Rosa Castro-Murillo, Flor De Jeanette Blas Bergara, Eduardo Franco-Chalco, Nataly Julissa Membrillo-Pillpe, Henry Castillo-Parra, Gabriela Samillán-Yncio, Laryn Smith

**Affiliations:** 1 Faculty of Health Science, Universidad María Auxiliadora, Lima 15408, Peru; rob.zegarra@gmail.com; 2 Academic Department of Nursing, Faculty of Medicine, Universidad Nacional Mayor de San Marcos, Lima 15001, Peru; gsamillani@unmsm.edu.pe; 3 Mental Health and Psychiatry Service, Hospital Hermilio Valdizan, Lima 150116, Peru; rosacastromurillo@gmail.com; 4 Epidemiology and Environmental Health Unit, Hospital San Juan de Lurigancho, Lima 15538, Peru; flordejeanetteblas@gmail.com; 5 Psychology Academic Department, Pontificial Catholic University of Perú, Lima 15008, Peru; eduardo.franco@pucp.edu.pe; 6 College of Nursing, Universidad Científica del Sur, Lima 15311, Peru; n.membrillo.pillpe@gmail.com; 7 Faculty of Psychology, Universidad de San Buenaventura, Medellín 050021, Colombia; gerencia@neuromind.net; 8 Nursing Doctoral Program, Faculty of Medicine, Universidad Nacional Mayor de San Marcos, Lima 15001, Peru; laryn12@gmail.com

**Keywords:** resilience, stress, psychometric study, nurses, empathy, CD-RISC-10, confirmatory factor analysis

## Abstract

**Background**: This study aims to psychometrically validate the abbreviated version of the Connor–Davidson Resilience Scale (CD-RISC-10) in Peruvian nurses, evaluating its convergent validity through its association with perceived stress and empathy. **Methods**: A cross-sectional psychometric study was conducted in 374 Peruvian nurses to evaluate the psychometric properties of CD-RISC-10 through confirmatory factor analysis (CFA). Furthermore, concurrent validity was assessed through correlational analysis using Spearman’s rho coefficient to evaluate the relationships among resilience, perceived stress, and empathy. **Results**: The CFA supported the predominantly one-dimensional model showing an adequate fit when the residual covariance between Items 4 and 7 was specified after correlating the residuals of Items 4 and 7 (CFI = 0.978, TLI = 0.971, RMSEA = 0.080, and SRMR = 0.044). Ordinal Cronbach’s alpha of 0.89 and McDonald’s omega of 0.81 were obtained. Concurrent validity showed significant correlations with perceived stress (rho = −0.53, *p* < 0.001) and empathy (rho = 0.31, *p* < 0.001). **Conclusions**: The CD-RISC-10 has adequate psychometric properties in Peruvian nurses. Future studies are needed to evaluate its factorial invariance between clinical specialties and establish normative thresholds.

## 1. Introduction

Resilience is currently recognized as an essential component for the sustainability of professional practice in nursing, particularly in clinical contexts characterized by high uncertainty, work overload, and continuous exposure to human suffering [1,2,3]. Strengthening nurses’ psychological resilience not only contributes to preserving the psychological integrity of nurses but also has a direct influence on patient safety and the technical and humanistic quality of the care provided [4,5,6]. From a theoretical perspective, resilience is defined as a dynamic, multidimensional, and continuous process by which people manage to maintain adaptive and healthy functioning, even in the face of adverse experiences or significant stressors [7,8].

The literature highlights that this construct is associated with various personal and contextual factors, including self-efficacy, positive coping, social support, and, in particular, empathy, which is considered a key resource that interacts synergistically with the resilient capacity to promote workplace well-being and empathy [9,10,11,12]. On the other hand, resilience has been identified as a protective moderating factor against indications of psychological distress, showing negative associations with perceived stress, anxiety, depression, and the different components of burnout [13,14,15].

The growing interest in assessing resilience in the nursing discipline has driven the development of various psychometric instruments that differ in their extent, level of specificity, and target population [4]. Internationally, the evidence reflects a constant evolution in its adaptation and validation processes. In 2016, in the United States, Mealer et al. performed the first adaptation of the Connor–Davidson Resilience Scale (CD-RISC) in intensive care unit nurses, proposing an abbreviated version of 16 items [16]. Three years later, in Iran, the Emergency Nurse’s Professional Resilience Tool (ENPRT) was designed, consisting of 37 items and aimed at measuring professional resilience in emergency services nurses, using a mixed approach of development and validation [6]. That same year, in China, the Resilience Scale-14 (RS-14) was validated in a sample of nurses with less than three years of experience, in order to explore their integration during the transition to clinical practice [17].

Subsequently, in 2022, the eight-item Nurse Team Resilience Scale (NTRS) was developed in China, aimed at assessing the collective resilience of nursing teams in the face of public health emergencies [18]. In 2023, they abbreviated the Wagnild & Young Resilience Scale into a 13-item version for application in U.S. nurses [19]. More recently, new cultural adaptations of the ENPRT in China [1] and the Persian version of the NTRS for resuscitation teams in Iran were reported [2]. Finally, in 2023, the validity and unifactorial structure of the 10-item CD-RISC were confirmed in nurses working in hospitals in Greece [5].

The CD-RISC has established itself as the most widely used and internationally recognized instrument for resilience assessment, thanks to its strong psychometric properties [20], factorial invariance, and generalization capacity to different clinical and population contexts [21]. In Spain, the abbreviated version of 10 items in Spanish was first validated with young university students [22] and older adults [23]. In Colombia, the validity and reliability of the short version in patients with chronic diseases were demonstrated [24] and later in the university population [25]. Along the same lines, Spanish research confirmed its unifactorial structure in multi-occupational samples [26] and established normative scales for the full 25-item scale [27].

In the Peruvian context, the CD-RISC has been the subject of psychometric analyses that demonstrate its cultural relevance and applicability in different groups. The first adaptations included translation and validation into the Quechua language in women affected by the armed conflict in Ayacucho [28]. Subsequently, they examined the 10-item version in university students, proposing an abbreviated version of seven items (CD-RISC-7) with superior adjustment indicators [29]. More recently, the CD-RISC-10 has shown adequate properties in vulnerable populations such as adolescent mothers [30], and the factorial validity of the 25-item South American version has been confirmed (CD-RISC-25SA) in adolescents, supporting a stable four-factor structure consistent with the regional sociocultural reality [8].

Although the CD-RISC has demonstrated validity in Peru among university students and adolescent mothers [29,30], there is currently no research available that psychometrically validates the existing Spanish version of this scale for Peruvian nursing professionals, a population subject to distinct care-related challenges. This gap is especially significant, as the lack of instruments with psychometric validation impedes reliable assessment of resilience, hinders analysis of its association with other psychosocial variables, and limits its integration into occupational health interventions. Therefore, the objective of the present study was to psychometrically validate the 10-item version of the CD-RISC in Peruvian nurses, evaluating its convergence validity via its association with perceived stress and empathy.

## 2. Materials and Methods

### 2.1. Sample and Procedures

A cross-sectional psychometric study was implemented with the participation of 374 Peruvian nurses to carry out the validation and determination of the psychometric properties of the CD-RISC-10 in the Peruvian context using Confirmatory Factor Analysis models. Additionally, concurrent validity was examined through correlational analyses between resilience, stress, and empathy. The study was carried out between June 2025 and February 2026.

After obtaining formal authorization from the original authors [7], a panel of experts was convened [31]. This panel consisted of eight specialist nurses (in mental health, emergency, and intensive care) and a psychologist. The group represented a multioccupational perspective. The objective of this panel was to evaluate the clarity and lexical comprehensibility of the items translated into Spanish, with the aim of adapting them to the professional context of nursing staff. The session, conducted via the Zoom platform (https://www.zoom.com/), began with the presentation of the objectives and a brief discussion about the definition of resilience and scale indicators to provide the conceptual framework and structure of the CD-RISC-10. Each item was then presented to the experts, allowing them to express their agreement or disagreement and to provide specific observations or alternative suggestions for reformulation when necessary. Following group analysis, modifications were made, and the revised item was submitted for approval through a vote. This analysis facilitated the approval of lexical adjustments in five items, replacing neutral Spanish expressions with reformulations frequently used by Peruvian nurses. In all cases, semantic and perceptual equivalence with the original item was maintained. The remaining five items were validated without modification. The modified version of the instrument was later reviewed by the research team and approved by the original author of the scale, after a back-translation process.

The five items modified during the expert panel are not shown in this manuscript because the scale items are protected by copyright. Their use and distribution are subject to authorization by the copyright holders. Researchers who wish to use the scale in the future may request authorization directly from the original authors.

The final sample consisted of Peruvian nurses who work in healthcare facilities such as public/private hospitals and clinics. The inclusion criteria were: (a) working in clinical areas of the healthcare facility, (b) having an electronic device with internet access, and (c) residing in Peru. Those who performed only administrative tasks or resided outside the country were excluded.

The minimum sample size was calculated considering a minimum of 10–20 subjects per item (n = 100–200) [32], prioritizing >300 to enhance factorial stability in CFA with moderate samples. Non-probabilistic convenience sampling was used until 374 valid responses were reached, which increases statistical power and mitigates individual biases.

Data collection was carried out virtually using the AllCounted platform (https://www.allcounted.com/) because of its ability to record response time and prevent random responses. The invitation was personally addressed to nurses who were pursuing postgraduate studies at Peruvian universities and professionals from verified health establishments, requesting a telephone number or email to send the unique link. Thus, compliance with selection criteria was verified through manual review of sociodemographic data.

All participants provided electronic virtual informed consent prior to the start of the questionnaire. The digital form began with the consent form (nature of the study, voluntariness, anonymity, risks/benefits), followed by a dichotomous acceptance question. Next, sociodemographic data (age, sex, residence, type of establishment and professional experience) were recorded before the instrumental scales.

### 2.2. Measurement Tools

#### 2.2.1. Resilience

The CD-RISC-10, a Spanish version authorized directly by the original authors, was used [7].

#### 2.2.2. Empathy

To assess convergence validity, the “Empathy” subscale of the Communication Skills Scale (CSS) was used, adapted and validated specifically for Peruvian nurses through confirmatory factor analysis (CFA), focus group, and cultural adjustment [33]. This subscale showed high reliability (McDonald’s ω = 0.82), factor loads > 0.62 and stable factor structure equivalent to the original Spanish version [34].

#### 2.2.3. Stress

The Perceived Stress Scale (PSS) was administered and structurally validated among Peruvian nurses using exploratory structural equation modeling (ESEM). The analysis revealed an optimal bifactorial structure, factor loadings of at least 0.50, and satisfactory reliability for both PSS-10 and PSS-14 [35].

Both scales used a 5-point Likert format (1 = strongly disagree; 5 = completely agree), administered in fixed order to minimize fatigue effects.

### 2.3. Analysis

The statistical analyses were conducted using the R v 4.5.3 computing environment and the RStudio v2026.01.2 interface. The *psych* v 2.6.3 package was used for descriptive and psychometric analyses, *lavaan* v 0.6-21 for structural equation modeling, and *semTools* v 0.5-8 for complementary fit and reliability analyses. In the first stage, the sociodemographic characteristics of the sample were examined by calculating frequencies and percentages for categorical variables. Then, evidence of validity based on internal structure was evaluated through Confirmatory Factor Analysis (CFA) using the Weighted Least Squares Mean and Variance adjusted (WLSMV) estimator, given the ordinal nature of the items. Model adequacy was assessed using comparative and absolute goodness-of-fit indices, considering CFI and TLI values greater than 0.95 and RMSEA and SRMR values below 0.08 as indicators of acceptable fit. As the original model’s fit indices fell below established benchmarks, modification indices were reviewed. This analysis supported the respecification of the model by permitting correlations between residual errors that are theoretically justified. Finally, score reliability was estimated using ordinal Cronbach’s alpha and McDonald’s omega coefficients, and the Average Variance Extracted (AVE) was calculated. Concurrent validity was examined using Spearman’s correlation coefficients between resilience, perceived stress, and empathy, following the assessment of normality assumptions using the Shapiro–Wilk test.

## 3. Results

The final sample consisted of 374 Peruvian nurses (age M = 39.77 years, SD = 9.11; range: 21–69 years), of which 88.24% were women. Regarding professional experience, almost half (47.33%) reported more than 10 years of work experience. Geographically, Metropolitan Lima predominated (74.06%), and 62.88% worked in hospitals (Table 1).

### 3.1. Evidence of Factorial Validity

**Figure 1:**
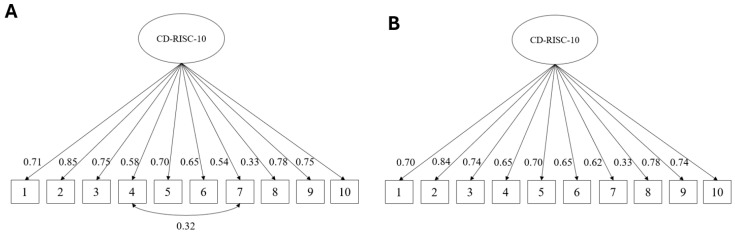
Initial and updated confirmatory factor models of the CD-RISC-10 in Peruvian nurses. Note. Panel (**A**) presents the initial one-factor model without correlated residuals, whereas Panel (**B**) presents the updated model including a correlated residual between Items 4 and 7. Standardized estimates are displayed. All factor loadings were statistically significant (*p* < 0.001). The correlated residual was added after inspection of modification indices and is interpreted cautiously, as both items share similar terminology related to functioning under pressure.

To examine evidence based on the internal structure of the CD-RISC-10, a Confirmatory Factor Analysis (CFA) was conducted in the sample of Peruvian nurses. First, a one-factor model without correlated residuals was tested (Figure 1, Panel A). This initial model showed suboptimal fit, with a significant chi-square value, χ^2^(35) = 241.28, *p* < 0.001. Although the SRMR (0.059) and CFI (0.945) approached acceptable values, the TLI (0.929) and RMSEA (0.126) indicated misfit.

Given these results, modification indices were inspected and suggested a residual covariance between Items 4 and 7. A revised one-factor model was subsequently estimated to incorporate this parameter (see Figure 1B). This modification was deemed conceptually plausible as both items pertain to effective functioning under pressure and may represent shared terminology as well as a distinct facet of self-regulation in stressful contexts, beyond the overarching resilience construct. The updated model demonstrated improved fit: χ^2^(34) = 114.86, *p* < 0.001, with CFI = 0.978, TLI = 0.971, RMSEA = 0.080, and SRMR = 0.044. Figure 1 presents both the initial and updated models. In the updated solution, all standardized factor loadings were statistically significant (*p* < 0.001) and ranged from 0.33 to 0.85. Item 2 showed the highest loading, followed by Items 8 and 9, whereas Item 5 showed the lowest loading. In addition, the residual covariance between Items 4 and 7 was positive and statistically significant (r = 0.32). Overall, these findings support a predominantly one-dimensional structure of the CD-RISC-10 in this sample, although the correlated residual should be interpreted cautiously and confirmed in future studies.

### 3.2. Evidence of Reliability by Internal Consistency

The reliability of the scale was analyzed through two internal consistency coefficients to ensure the accuracy of the measurement. An Ordinal Cronbach’s Alpha coefficient of 0.89 was obtained, indicating a high homogeneity between the instrument’s items. In addition, the McDonald’s Omega coefficient was 0.81, suggesting an adequate internal consistency for the use of the scale in research and professional practice contexts.

### 3.3. Average Variance Extracted (AVE)

Regarding the Average Variance Extracted (AVE), the estimated value was 0.46, which is slightly below the conventional reference value of 0.50. The findings indicate that the latent resilience factor accounted for a moderate, albeit suboptimal, proportion of the shared variance among the CD-RISC-10 items within this sample of Peruvian nurses. Therefore, the AVE should be interpreted with caution, as it provides only limited support for common variance explained by the factor and indicates a potential area for further examination in future studies.

### 3.4. Evidence of Concurrent Validity

Table 2 presents the descriptive statistics, Shapiro–Wilk normality test, and Spearman correlation coefficients for resilience, perceived stress, and empathy. The median scores were 4.00 (IQR = 0.70) for resilience, 2.43 (IQR = 0.57) for perceived stress, and 4.80 (IQR = 1.40) for empathy. The Shapiro–Wilk test indicated significant deviations from normality for all three variables (resilience: W = 0.98, *p* < 0.001; perceived stress: W = 0.99, *p* = 0.005; empathy: W = 0.95, *p* < 0.001). Accordingly, nonparametric correlations were estimated using Spearman’s rho. Resilience was significantly and negatively correlated with perceived stress (rho = −0.53, *p* < 0.001), and significantly and positively correlated with empathy (rho = 0.31, *p* < 0.001). Furthermore, empathy showed a significant negative correlation with perceived stress (rho = −0.23, *p* < 0.001). The findings demonstrate that the relationships between the study variables aligned with anticipated patterns.

## 4. Discussion

This study offers evidence supporting a predominantly one-dimensional structure of the CD-RISC-10 among Peruvian nurses. The unifactorial model was confirmed by confirmatory factor analysis (CFA), and convergent validity was established through its correlation with perceived stress and empathy.

These findings contribute to the global body of evidence on the abbreviated version of this scale in the Peruvian sociocultural context, where it lacked specific validation in nursing professionals. The results converge with recent international validations in nursing personnel, such as the study carried out in Greece, where a one-dimensional structure with excellent reliability was also reported [5]. Likewise, studies involving Spanish professionals across various occupations have affirmed the reliability of this single-factor model [26]. Although other instruments, such as the RS-14, have shown two-factor structures in Eastern nurses [17], the 10-item version used in this study showed acceptable psychometric behavior in this sample.

As for reliability, the CD-RISC-10 showed adequate internal consistency in this sample, with an ordinal Cronbach’s alpha of 0.89 and a McDonald’s omega of 0.81. These values are consistent with findings from prior research, indicating that the items reliably serve as indicators of the construct [5,21]. In comparative terms, the reliability observed here was slightly higher than that reported by Serrano et al. (α = 0.81) [23] and Soler et al. (α = 0.87) [26] and somewhat lower than that reported by Wang et al. (α = 0.91) [36]. Regarding the Average Variance Extracted (AVE), the value obtained (0.46) was slightly below the conventional reference threshold of 0.50. This indicates that while the latent factor accounted for a significant portion of the shared variance among the items, the degree of common variance explained was suboptimal. Therefore, this finding should be interpreted cautiously and considered a limitation that warrants further examination in future studies [21,37].

The analysis of concurrent validity showed a significant and moderate negative association between resilience and perceived stress, indicating that higher resilience was related to lower levels of perceived stress in this sample of Peruvian nurses. This pattern aligns with established theoretical frameworks and prior research, which characterizes resilience as a personal asset linked to improved psychological adjustment in challenging occupational settings [7,21]. This finding is consistent with the Job Demands–Resources (JD-R) model, which conceptualizes resilience as a protective personal resource that may help buffer the impact of occupational demands on psychological distress and burnout [3,13,38]. Accordingly, the identified relationship with perceived stress serves as substantiating evidence for the concurrent validity of the CD-RISC-10, demonstrating consistency with the established theoretical framework of resilience [14,15].

Likewise, resilience was positively and significantly associated with empathy, suggesting that nurses with higher resilience scores also tended to report higher levels of empathic disposition. This finding is also consistent with previous research indicating that resilience is positively linked to interpersonal and emotional resources in healthcare professionals [3,10]. Previous research involving nurses indicates that empathy and resilience interact as valuable personal resources, contributing to enhanced professional quality of life and reduced levels of compassion fatigue [10,12]. Accordingly, the positive association identified in this study offers further corroboration of concurrent validity, demonstrating that the CD-RISC-10 correlates with empathy as anticipated. However, given the cross-sectional nature of the data, these findings should be interpreted as associative rather than causal [4,9,39].

Among the practical implications of this study, the findings suggest that resilience is a relevant personal resource associated with lower perceived stress and higher empathy in Peruvian nurses. From a practical standpoint, these findings highlight the value of incorporating resilience assessments into comprehensive strategies designed to monitor occupational well-being and determine professional support needs in high-demand clinical settings. Resilience, empathy, and perceived stress are pertinent psychosocial variables associated with professional shifts within nursing environments. At the educational and organizational levels, these results suggest that nursing training and continuing professional development could benefit from including content related to adaptive coping, emotional regulation, and stress management. Likewise, interventions aimed at strengthening resilience and preserving empathic capacities may be relevant for promoting occupational well-being and supporting the quality of care in demanding healthcare environments.

First, due to the cross-sectional and correlational design, the findings do not allow causal inferences regarding the relationships among resilience, perceived stress, and empathy. Second, the use of a non-probabilistic sample limits the generalizability of the results to other nursing populations and professional contexts. Third, the online recruitment procedure may have introduced selection bias, as participation was limited to nurses with internet access and willingness to complete an online survey. Fourth, although the respecified CFA model showed improved fit, this solution depended on a correlated residual between Items 4 and 7, which was introduced after inspection of modification indices and therefore should be interpreted cautiously until replicated in independent samples. Fifth, because the sample was predominantly composed of women, which reflects the demographic composition of the nursing profession, men are underrepresented. Consequently, the results should be interpreted with caution when extrapolating them to male nurses. Similarly, most participants from the country’s capital, Lima, who work in hospital institutions, limit the applicability of the findings to rural contexts, populations in Andean areas, the Amazon, and those who work in primary care facilities. In future studies, it is recommended to recruit more heterogeneous samples and formally evaluate the measurement invariance according to sex, geographic scope, and between nursing specialties and levels of health care to determine if the CD-RISC-10 works equivalently in these different subgroups of Peruvian nursing professionals. Finally, no longitudinal data were available to examine temporal stability indicators such as test–retest reliability or intraclass correlation coefficients, which should be addressed in future validation studies.

## 5. Conclusions

This study provides supportive psychometric evidence for the use of the CD-RISC-10 in a sample of Peruvian nurses. The findings were consistent with a predominantly one-dimensional structure, exhibited adequate internal consistency, and demonstrated expected associations with perceived stress and empathy. Collectively, these findings indicate that the CD-RISC-10 is a potentially valuable tool for efficiently assessing resilience within this professional population. Future studies should continue examining its internal structure, temporal stability, and factorial invariance across nursing subgroups. Until measurement invariance is formally established, researchers should exercise caution when comparing resilience scores across sex, geographic regions, or care levels and examine their applicability in other healthcare settings.

## Figures and Tables

**Table 1:** Sociodemographic and job-related characteristics of the sample.

	*f*	*%*
Sex		
Male	44	11.76
Female	330	88.24
Years of experience		
Less than 5	84	22.46
Between 5 and 10 years	113	30.21
More than 10 years	177	47.33
Department of residence		
Lima	277	74.06
Other departments of Peru	97	25.94
Work Environment		
Hospital	227	62.88
Health center/Health post	59	16.34
Other *	75	20.78

* Clinic, independent practice and others.

**Table 2:** Descriptive statistics, normality test and Spearman’s correlation matrix between Resilience, Perceived Stress and Empathy.

			Shapiro–Wilk’s Test		Spearman’s rho		
	Med	IQR	W	*p*	1	2	3
1. Resilience	4.00	0.70	0.98	<0.001	-		
2. Perceived stress	2.43	0.57	0.99	0.005	−0.53 ***	-	
3. Empathy	4.80	1.40	0.95	<0.001	0.31 ***	−0.23 ***	-

*** *p* < 0.001.

## Data Availability

The information will be available upon request, addressed to the corresponding author. Public access is restricted because the scale items are protected by copyright, and their use and distribution are subject to authorization by the copyright holders.
